# Effect of Different Endodontic Sealers on the Push-out Bond Strength of Fiber Posts

**DOI:** 10.7508/iej.2016.02.009

**Published:** 2016-03-20

**Authors:** Mohammad Forough Reyhani, Negin Ghasemi, Saeed Rahimi, Amin Salem Milani, Elnaz Omrani

**Affiliations:** a *Department of Endodontics, Dental School, Tabriz University of Medical Sciences, Tabriz, Iran; *; b * Dental and Periodontal Research Center, Department of Endodontics, Dental School, Tabriz University of Medical Sciences, Tabriz, Iran; *; c * Dental School, Tabriz University of Medical Sciences, Tabriz, Iran*

**Keywords:** Bond strength, Resin Cement, Root Canal Sealer

## Abstract

**Introduction:**

The aim of this *in vitro *study was to evaluate the effect of MTA-based sealer (MTA Fillapex), eugenol-based sealer (Dorifill) and an epoxy resin sealer (AH Plus) on the bond strength of fiber posts cemented with a self-etch adhesive.

**Materials and Methods:**

The root canals of 72 maxillary incisors were prepared using the step-back technique after removing/cutting off the crowns. The samples were randomly divided to 4 groups (*n*=18). In group 1 (the controls) gutta-percha was used without sealer. In groups 2, 3 and 4, the canals were filled with gutta-percha using AH Plus, Dorifill and MTA Fillapex sealers, respectively, by cold lateral compaction technique. After post space preparation, the fiber posts were cemented in the root canals using self-etch adhesive. Then 1-mm-thick disks were prepared from the coronal thirds of all the root canals and subjected to a push-out test. Data were analyzed using the one-way ANOVA and post hoc Tukey’s tests.

**Results:**

The maximum (4.45±0.09 MPa) and minimum (1.02±0.03 MPa) bond strength values were recorded in the control and Dorifill groups, respectively. The mean push-out bond strength values were similar for MTA Fillapex and AH Plus sealers (*P*>0.05). However these values were significantly higher than that of the Dorifill sealer (*P*<0.05).

**Conclusion:**

Sealer type affected the bond strength of the fiber posts and MTA Fillapex decreased the dislodgment resistant of the fiber post.

## Introduction

Radicular posts are suggested for retention of the final restoration during the reconstruction of endodontically treated teeth with non-sufficient coronal structure [1]. Fiber posts have low rigidity, their elasticity is more compatible with dentin and they can form a single unit with the surrounding root. On the other hand, they exhibit favorable stress distribution; therefore they are advocated because they result in less root fracture [1, 2]. Resin cements are commonly used for bonding of posts to the root canal walls. Fiber posts have excellent bonding properties to resin cements and dentin [2].

The self-etch adhesive systems do not require pretreatment of the root dentine; therefore, they offer a simpler bonding process [3].The bonding mechanism in self-etch adhesive systems depends on chemical bonding and micromechanical retention [4]. Studies have shown that self-etch adhesive systems exhibit high bond strength and good marginal adaptation with dentin [5].

During cementation of posts, the bond between the resin cement and the root dentin might be affected by the type of the endodontic sealer used for root canal obturation and can finally affect the retention of the post [6].

Eugenol-based cements are still very popular among dentists and researchers, due to their long history of use [7, 8]. A large number of comparative studies have shown that eugenol-containing cements have a negative effect on the strength of fiber posts cemented with resin cements because eugenol interferes with the polymerization process [6, 9]. On the other hand, some studies have shown that eugenol-containing and non-eugenol sealers have a similar effect on the retention of posts cemented with resin cements because these sealers do not penetrate deep into the dentinal tubules [10]. 

AH Plus (Dentsply DeTrey, Konstanz, Germany) is an epoxy resin, which has a proper performance [11]. Researchers have introduced it as a gold standard for the comparison of all the sealers and filling materials to be bonded in the root canal [12].

A study showed a similar effect of AH Plus sealer and eugenol-based sealer (Endofill) on the bond strength of fiber posts cemented with resin cement, with no statistically significant differences [7].

Recently, MTA-based sealers are introduced to achieve the biologic properties and proper seal of MTA. One of these sealers is MTA Fillapex, which is marketed as two pastes. Based on manufacturer’s report, the sealer contains MTA, salicylates resin, natural resin, silica and bismuth after mixing. Based on the results of a study, this sealer releases calcium and creates an alkaline environment. Highly alkaline materials help mineralize hard tissues, in addition to their antimicrobial properties [13].

A recent study showed that the bond strength of MTA Fillapex to root dentin is similar to that of AH Plus [14]. However, there is no data available on the effect of MTA-based sealers on the bond strength of fiber posts. Therefore, the aim of this *in vitro* study was to evaluate the effect of an MTA-based sealer on the bond strength of fiber posts cemented with a self-adhesive resin cement in comparison with a eugenol-containing sealer (Dorifill) and an epoxy resin sealer (AH Plus). The null hypothesis was that the type of root canal sealer had no effect on the dislodgment resistance of the fiber post.

## Materials and Methods

A total of 72 single-rooted maxillary incisors, extracted for periodontal reasons, were selected for this study. Teeth with caries, curved roots, cracked roots, open apices or previous endodontic treatment were excluded from the study. The teeth were cleaned of any soft tissue remnants and stored in 0.5% chloramine-T solution until the study. The tooth crowns were removed at cementoenamel junction (CEJ) level using a diamond disk (SP 1600 Microtome, Leica, NuBlock, Germany) to leave a root length of 14 mm.

The patency of the root canals was confirmed with a #10 K-file (Mani Inc., Japan) and the working length was determined 1 mm shorter than the file length after its emergence from the apical foramen. The root canals were prepared using the step-back technique. All the root canals were prepared up to #40 as the master apical file by one operator and the coronal and middle thirds of the root canals were enlarged using #4, 3 and 2 Gates-Glidden drills (Mani Inc., Japan). During the root canal preparation procedures, the irrigation protocol consisted of 2.5% NaOCl irrigation during instrumentation and a final flush with normal saline at the end of preparation procedures, followed by a 5-min use of 17% ethylenediaminetetraacetic acid (EDTA) (Pulpdent Corporation, Watertown, MA, USA). After the final rinse with normal saline, the root canals were dried with paper points and were randomly assigned to 4 groups of 18 and obturated with gutta-percha (Aryadent, Tehran, Iran) using the cold lateral compaction technique. In group 1 (the control) gutta-percha was used without sealer. In groups 2, 3 and 4, AH Plus resin-based sealer (Dentsply DeTery, Konstanz, Germany), Dorfill zinc oxide-eugenol sealer (Dorident Company, Austria) and MTA Fillapex (Angelus Solucoesodontologica, Londrina, PR, Brazil) were used for root canal obturation, respectively. The sealers were used according to manufacturers’ instructions. Cavit (3M ESPE, Seefeld, Germany) temporary restoration material was used for the coronal seal of the access cavity. The specimens were stored at 37^º^C under 100% relative humidity for 7 days.

***Post space preparation and cementation of the posts***

Gutta-percha was removed from the root canals with #2 Peeso reamers (Mani Inc., Japan) to leave at least 4 mm of gutta-percha at the apical third of the root canal. The post space was prepared up to a depth of 9 mm using the blue drill of the post system (Innopost Premier Anatomic; Innotech, Italy). The post space was irrigated with distilled water and dried with paper cones. The self-adhesive resin cement (Clearfil SA Luting, Kuraray, Osaka, Japan) was placed in the root canals using the special applicator and the external surface of the #1.1 post was cleaned with alcohol. Equal lengths of the two pastes of the resin cement were placed on a glass slab and mixed for 10 sec and then placed in the root canal. The posts were placed completely in the root canal and seated with finger pressure. Finally, the resin cement was polymerized for 20 sec using an LED light-curing unit. The tip of the conductor of the light-curing unit was placed at canal orifice. 

***Push-out test***

Twenty-four hours after storing the specimens at 37^º^C under 100% of relative humidity, a diamond disk (Isimet, Buehler Ltd, Lake Bluff, IL) was used to prepare transverse 1-mm-thick sections from the coronal third of the specimens in the form of disks. The push-out test was carried out using the special jig of the push-out test equipment. The samples were fixed on the jig with cyanoacrylate glue (Mad Wolf, Elkim Eektro Kimya San. Tic. A.S. Istanbul, Turkey). It was ensured that the coronal surface was in contact with the instrument and the post was at the center of the space between the metallic supports. The cylindrical plunger, measuring 0.9 mm in diameter, was placed at the center of the post, perverting its contact with the surrounding dentin. The amount of push-out force applied by the tip of the cylindrical plunger was measured by a universal testing machine (Instron, Model 1334, Instron Corp, Cauton, MA). The tip of the cylindrical instrument was placed on the surface of the fiber post and the force was applied at a crosshead speed of 1 mm/min in the apico-coronal (considering the tapered fiber post) direction. The maximum force necessary to push the fiber out of the sample was considered as the bond failure point and was recorded by using the following formula: *A=2πr×h*, where *r* is the radius of the root canal space and *h* is the thickness of the samples in mm. Therefore, the bond strength (*δ*) was calculated in MPa using the following formula: *δ=F/A*

***Statistical analysis***

After the analysis of descriptive data (means±standard deviations) of bond strength values in different study groups, one-way ANOVA was used to evaluate the effect of the sealer type on the bond strength of fiber posts. The post hoc Tukey’s tests were used for two-by-two comparisons of the groups. SPSS software (Statistical Package for Social Science, SPSS, version 18.0, SPSS, Chicago, IL, USA) was used for statistical analyses and the level of significance was set at 0.05. 

## Results

The maximum and minimum bond strength values were recorded in the control and Dorifill groups, respectively. The sealer type had a significant effect on the bond strength (*P*=0.02). Two-by-two comparisons of the groups showed significant differences in bond strength values between the control group and the other groups (*P*=0.03). Resistance to dislodgment in the MTA Fillapex and AH Plus group fiber posts was significantly higher than that in the Dorifill group (*P*=0.001), with no significant differences between the MTA- and resin-based sealers (*P*=0.8). 

## Discussion

The final aim of researches on dental materials is to achieve a real bond between the different dental materials and between the dental materials and the tooth structure. It is obvious that no material is used alone to fill any space in teeth; rather, the material should have specific properties, including adhesion, sealing, biocompatibility and the ability to create a durable bond in the versatile oral cavity environment [15].

**Table 1:** The mean±SD of the push-out bond strength (Mpa) of study groups

**Sealer**	**Bond Strength**
**MTA Fillapex**	2.24 (0.02)
**AH Plus**	2.08 (0.02)
**Dorifill**	1.02 (0.03)
**Control **	4.45 (0.09)

Fiber posts are proper alternatives to metallic posts, which can form a uniform complex with the root canal walls after being cemented with resin cements. In other words, their retention depends on the interface between root dentin, cement and post surface [16]. Debonding mainly occurs at these sites [17]. Therefore, the success of fiber post cementation depends on the proper bond to the root canal walls [16]. Studies have shown that cementation of fiber posts with resin cements results in better retention, less microleakage and more resistance to tooth fracture [7]. The main reason for using resin adhesive systems is their greater retention compared to zinc phosphate cement. In addition, the retention of a dowel which is cemented passively with a resin cement is the same as that of a dowel placed actively. The other advantage of resin cements is their lower microleakage [18].

The bond strength of post-cement-dentin interface is influenced by several factors, including a decrease in reaction of the luting agent with dentin, polymerization rate, stress resulted from polymerization shrinkage of the resin luting agent, the presence of endodontic sealer or gutta-percha remnants, the configuration of the root canal and differences in the density and orientation of dentinal tubules in different areas of the root dentin [19]. The bond strength can be measured using different techniques, including conventional shear, microtensile, pull-out and push-out tests. The push-out test which uses a shearing stress at the dentin-post-cement interfaces, simulates the clinical conditions. Although the conventional shear and microtensile tests are extensively used, based on some reports, the push-out test provides a better estimate of resistance to dislodgment and is more reliable due to the absence of premature failure and a diversity in the distribution of data [20].

Different variables are considered as the confounding factors during the push-out test, including the ratio of the diameter of the plunger to the dimension of the prepared root canal. Based on previous studies when the diameter of the plunger is 70-90% of that of the root canal, the effect of this confiding factor is minimal [21]. In the present study the 1.1-mm diameter of the root canal was proportional to the diameter of the #4 Gates-Glidden drill which was used for preparation of the coronal zone of the root canal and the diameter of the plunger was 0.9 mm. Another confounding factor is the thickness of the prepared disks, which was 1 mm and is considered as approximate for decreasing the sliding friction of the metallic plunger. The plunger was perpendicular to the surface of the samples, which was verified by a triangle to minimize the effect of this confounding factor. Based on the results of previous studies, this angle should not be less than 10 degrees [21].

The results of the present study showed the highest bond strength values in the control group in which no sealer was used, consistent with the results of some other studies [8]. The most plausible explanation might be the fact that in the control group the patent orifices of dentinal tubules might allow the maximum penetration of resin cement. The majority of studies have shown that eugenol-containing cements might decrease the bond strength of resin cements [8, 22, 23]. In the present study, the minimum bond strength was recorded in the Dorifill group, as a eugenol-based sealer. The reason might be attributed to the remnants of eugenol on dentin; since eugenol has phenolic compounds, it increases the release of free radicals and interferes with the polymerization of resin cement. 

Elimination of eugenol remnants in the root canal might be necessary to improve the adhesive process. Izadi *et al*. [24] reported that a long interval between obturation of the root canal with a eugenol-based sealer and placement of a post might decrease the post retention due to the deeper penetration of eugenol into the dentinal tubules. Since eugenol-based sealers have a long setting time, phenol in its composition might penetrate deep into the root dentin. Tjan and Nemetz [25] reported that eugenol decreases the retention of posts cemented with Panavia, although use of alcohol or acid etching as intracanal irrigation might increase retention. In the present study, no preparation and irrigation were carried out with acid, which resulted in a decrease in bond strength in the eugenol group. 

AH Plus is a commonly used epoxy resin sealer used in association with gutta-percha. It has some properties such as low solubility and disintegration and is considered the gold standard because it has excellent sealing ability due to its proper adhesion [26]. In addition, in the present study the bond strength of AH Plus, was higher than that of Dorifill. Cohen *et al.* [27] reported that epoxy resin in the composition of resin-based cements, such as AH Plus, does not interfere with the activation of free radicals in composite resin. Therefore, the resin-based sealer has no reversing effect on the adhesion of resin cement. Cecchin *et al. *[28] reported that the high bond strength of resin-based sealers is due to the presence of epoxy resin in their composition, which is similar to the composition of the resin cement.

Recently, attempts have been made to modify the original formulation of MTA to improve its properties such as flow, setting time and adhesion, so that it can be used as an endodontic sealer [28-30]. One of these sealers is MTA Fillapex, which is marketed as two pastes and consists of MTA, salicylates resin, natural resin, silica and bismuth after mixing [14]. This sealer has high sealing ability, proper bactericidal activity and biocompatibility. Little information is available on its adhesion properties. Based on the results of the present study, the bond strength of this sealer with the fiber post is similar to that of AH Plus, which might be attributed to the resin structure of these two sealers and also to the fact that the reins in the structure of this sealer do not compromise the adhesion of fiber post to the root dentin [29]. 

The majority of root canal sealers contain eugenol, which has a negative effect on the polymerization of composite resins. In addition, eugenol-containing sealer is present in the root canal after post space preparation; as a result, it is advisable to use zinc phosphate cement instead of cements that form bonds when eugenol-containing sealers have been used during endodontic treatment [27]. However, the use of eugenol-containing sealers does not always result in a decrease in bond strength and it occurs only in cases in which a bonding agent is used that etches the smear layer but is not rinsed. In addition, if rinsing is carried out with ethyl alcohol or etching is carried out with 37% phosphoric acid, the post will have higher bond strength and will not be dislodged [18]. 

Similar studies on different techniques such as irrigation with acid or ultrasound/lasers before cementation of posts, are suggested.

## Conclusion

Based on the results of the present study, the type of the sealer used for root canal obturation can affect the bond strength of the fiber post with it’s resin cement.
